# Overview of Evidence of Antimicrobial Use and Antimicrobial Resistance in the Food Chain

**DOI:** 10.3390/antibiotics9020049

**Published:** 2020-01-28

**Authors:** Houda Bennani, Ana Mateus, Nicholas Mays, Elizabeth Eastmure, Katharina D. C. Stärk, Barbara Häsler

**Affiliations:** 1 Veterinary Epidemiology, Economics and Public Health Group, Department of Pathobiology and Population Sciences, Royal Veterinary College, Hawkshead Lane, North Mymms, Hatfield, Hertfordshire AL9 7TA, UK; amateus@rvc.ac.uk (A.M.); kstaerk@rvc.ac.uk (K.D.C.S.); bhaesler@rvc.ac.uk (B.H.); 2 Policy Innovation Research Unit, Department of Health Services Research & Policy, London School of Hygiene and Tropical Medicine, London WC1H 9SH, UK; Nicholas.Mays@lshtm.ac.uk (N.M.); Elizabeth.Eastmure@lshtm.ac.uk (E.E.); 3 SAFOSO AG, Waldeggstrasse 1, CH 3097 Liebefeld, Switzerland

**Keywords:** antimicrobial resistance, antibiotic resistance, antimicrobial use, food chain, integrated surveillance

## Abstract

Antimicrobial resistance (AMR) is a global health problem. Bacteria carrying resistance genes can be transmitted between humans, animals and the environment. There are concerns that the widespread use of antimicrobials in the food chain constitutes an important source of AMR in humans, but the extent of this transmission is not well understood. The aim of this review is to examine published evidence on the links between antimicrobial use (AMU) in the food chain and AMR in people and animals. The evidence showed a link between AMU in animals and the occurrence of resistance in these animals. However, evidence of the benefits of a reduction in AMU in animals on the prevalence of resistant bacteria in humans is scarce. The presence of resistant bacteria is documented in the human food supply chain, which presents a potential exposure route and risk to public health. Microbial genome sequencing has enabled the establishment of some links between the presence of resistant bacteria in humans and animals but, for some antimicrobials, no link could be established. Research and monitoring of AMU and AMR in an integrated manner is essential for a better understanding of the biology and the dynamics of antimicrobial resistance.

## 1. Introduction

Antimicrobial resistance (AMR) is recognized as one of the key threats to human and animal health at global level with significant economic implications [1,2]. Antimicrobials (AMs) include antibiotics, antivirals, antifungals and antiprotozoals. These are active substances of synthetic or natural origin, which kill or inhibit the growth of microorganisms [3]. Antimicrobial resistance refers to the ability of microorganisms, such as bacteria, to become increasingly resistant to an AM to which they were previously susceptible and, as a result, infections may persist in the body, increasing the risk of spreading to others [2,3]. Resistance to AMs can be acquired either by mutations in pre-existing or previously acquired genes or by horizontal gene transfer (HGT), which is the acquisition of new genes from other bacteria. The later mechanism is the main cause of resistance to AMs [4]. While AMR development is a naturally occurring phenomenon, the overuse and misuse of AMs can accelerate this process [5]. Exposure to AMs increases the selection pressure and mobilization of genes between bacteria [6]. Of particular concern is the emergence of resistance in Gram-negative bacteria, which constitutes a major public health risk due to reported resistance to carbapenems and colistin, which are considered last resort AMs for multi-drug resistant (MDR) bacteria, and the lack of new AMs to replace them [7,8]. Drug-resistant pathogens are estimated to be responsible for 25,000 deaths per year in the European Union (EU) and 700,000 deaths per year globally [3]. This number is estimated to reach 10 million deaths per year by 2050 if no action is taken [1]. Antimicrobial resistance is also responsible for an increase in the costs of treatments and decrease in labor productivity due to prolonged illness. In the EU, it is estimated that AMR costs EUR 1.5 billion annually in healthcare costs and productivity losses. At a global level, it is estimated that drug-resistant infections could have a cumulative cost to global economic output of USD 100 trillion by 2050 [1,3].

Antimicrobials are used to treat infectious diseases in humans and animals; in the latter, AMs are also used in a non-therapeutic way for disease prophylaxis (i.e., AMs administered to a herd or a flock at risk of a disease) and for metaphylactic purposes (i.e., AMs administered to healthy animals in a herd or flock of animals where some have already shown clinical signs of infection) [9]. Furthermore, they can also be used as growth promoters (AGPs) when used in sub-therapeutic dosages to increase productivity [10]. In the EU, the use of AGPs in animal feed and water was banned in 2006 as a response to increasing concerns about the effect of this type of use on resistance [11]. The large quantities of AMs used in animal production contribute to the development of AMR in animal populations [12,13,14].

Bacteria carrying resistant genes from animals can be transmitted to humans directly through the food chain by the consumption of inadequately cooked food and raw food, the handling of raw food, by cross-contamination with other foods, or indirectly through the environment. Resistant bacteria can also be transmitted directly from animals in farms [4,15]. There are concerns that widespread use of AMs in the food chain constitutes an important source of AMR, potentially affecting humans but the extent to which this use affects humans is not well understood.

The aim of this review was to examine the evidence regarding the links between antimicrobial use (AMU) in the food chain and the occurrence of AMR in people and animals. The inclusion of a wide range of studies from the scientific and grey literature using an integrative approach has allowed to produce a comprehensive review of interest to researchers, policy makers and practitioners in the field even though no formal technical assessment of the quality of the studies was conducted.

## 2. Results

**Figure 1:**
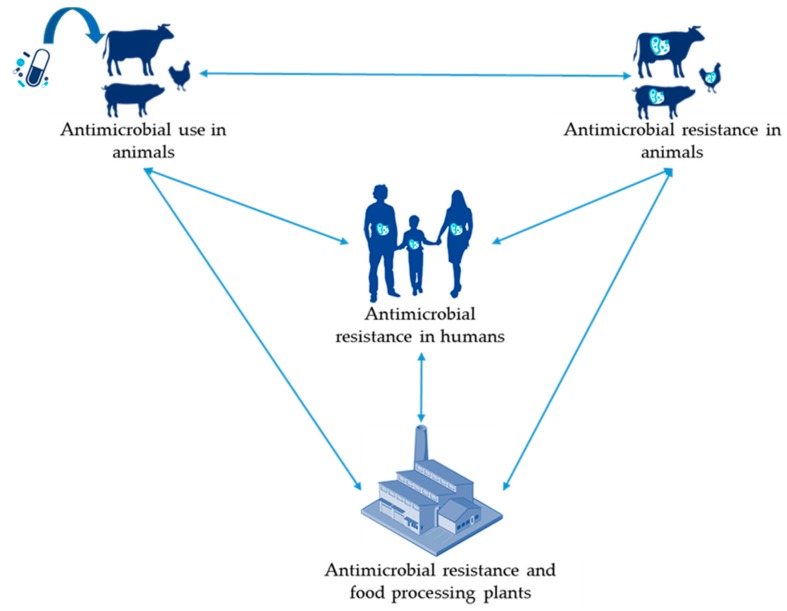
Links between different fields of data identified on the relationship between anti-microbial use (AMU) and anti-microbial resistance (AMR) in the food chain and people.

The studies included in this review are summarized in Table S1. In the following sections, we review the different links identified between AMU and AMR in the food chain and AMR in humans as illustrated in Figure 1.

### 2.1. Antimicrobial Use and Antimicrobial Resistance in Animals

The studies investigating links between AMU and AMR in animals were mainly national surveillance reports from countries where integrated surveillance has been conducted for a long time, namely Denmark, Sweden and the Netherlands, and reports of surveillance programs for AMR and AMU in the EU.

Available evidence suggests that there is a correlation between the quantity of AMs used in animals and the development of resistance in bacteria present in these animals. This link has been demonstrated for a range of combinations of specific pathogens, commensals, antimicrobial substances and livestock species, as detailed below.

The resistance of *Escherichia coli (E. coli)* to cefotaxime (a 3rd generation cephalosporin) in broilers in the Netherlands increased after 2003 to reach a level of more than 20% in 2007 (percentage of *E. coli* isolates resistant to cefotaxime); this resistance prevalence decreased sharply after the ban on the use of ceftiofur (also a 3rd generation cephalosporin) in hatcheries in 2010 to reach a level of 2.9% in 2014 [16]. For *Salmonella* isolates, resistance levels varied over the years. Antimicrobial use in food-producing animals (FPAs) has been reduced considerably in the Netherlands in recent years due to a government policy to reduce AMU after the country had been identified as one of the highest consumers of AMs among EU countries in 2007 [17]. Between 2007 and 2013, there was a 63% reduction in AMU in animals, from 565 tonnes per year to 217 tonnes per year, accompanied by a reduction in the levels of AMR in animals. A reduction in resistance was observed in commensal *E. coli* isolated from broilers, slaughter pigs and veal calves between 2009 and 2014, and a reduction of resistance in *E. coli* isolated from meat from poultry, beef, pork and veal. However, the data for *Salmonella* and *Campylobacter* did not show any reducing trend [16,18]. This may be due to the differences in resistance mechanisms expressed by these bacteria, and their ability to reverse —or not— to being susceptible once AMU is decreased. Some bacterial species are naturally resistant to certain antimicrobials (intrinsic resistance), which are mediated by chromosomal genes.

This type of resistance is most likely responsible for differences between different genera and species [19]. For example, Gram-negative bacteria generally have higher levels of intrinsic resistance to certain antimicrobial substances compared to Gram-positive, due to the composition and complexity of their inner cell wall, made of peptidoglycans, and an outer membrane composed of phospholipids and lipopolysaccharides (LPS). Resistance to penicillin G, expressed by most Gram-negative bacteria, is an example of this intrinsic resistance, as this antibiotic is not able to penetrate the outer layer [19].

Evidence from Denmark of the percentage of resistant isolates in indicator *E. coli* from healthy pigs, cattle and broilers between 2001 and 2008 showed that the highest level of resistance was found in pigs, which had the highest level of AMU compared to cattle and broilers in Denmark (resistance levels for streptomycin, sulphonamide and tetracycline ranged between 25% and 48%) [20]. Another study found a significant impact of the 2010 voluntary ban of cephalosporin use in Danish pig production on the prevalence of extended-spectrum cephalosporinase (ESC)-producing *E. coli* in pigs and pork. The occurrence of ESC-producing *E. coli* declined in pigs at slaughter from 11.8% in 2010 to 3.6% in 2011, and from 11% in 2010 to 0% in 2011 in pig farms [21]. In Belgium, a study exploring the association between AMU reduction (15.9% decrease between 2011 and 2015) and resistance of commensal *E. coli* between 2011 and 2015 showed positive correlations between AMR and the use of the corresponding antimicrobial class during the study period for most AMs (significant for ampicillin and borderline significant for colistin, sulfamethoxazole, trimethoprim and tetracycline); for chloramphenicol and gentamicin, the correlation was negative [22]. In a recent systematic review and meta-analysis, Tang et al. (2017) investigated the associations between restricting the use of AMs in FPAs and AMR in such animals and humans. The results showed that interventions to mitigate AMU in animals reduced AMR in bacteria in these animals, with an overall reduction in AMR by about 15% and MDR bacteria between 24% and 32%.

The use of different AM substances that belong to the same drug class can favor the survival of bacteria that harbor genes for the same resistance mechanism (cross-resistance). Avoparcin is a glycopeptide, previously used in veterinary medicine as an AGP, and belongs to the same class of AMs as vancomycin—a critically important AM for human medicine according to the World Health Organization (WHO) [23]. Denmark was one of the first countries to ban avoparcin as AGP in 1995 after discovering that its use selected for the occurrence of vancomycin-resistant *Enterococci* (VRE) [20]. The ban of avoparcin led to a marked decrease in VRE isolated from fecal samples of broilers, from 72.7% in 1995 to 5.8% in 2000 and <3% in 2005 [24,25]. Resistance to erythromycin (a macrolide) among *E. faecium* and *E. faecalis* isolates from pigs was almost 90% between 1995 and 1997; resistance levels decreased to 46.7% and 28.1% for *E. faecium* and *E. faecalis*, respectively, following a sharp decrease in tylosin (also a macrolide) use in 1998–1999 (the government banned the use of avoparcin in 1995 and virginiamycin in 1998 and producers voluntarily stopped all use of antibiotics for growth promotion at the end of 1999) [25].

A systematic literature review of publications from the United States, Canada and Denmark between 2010 and 2014 investigated the relationship between AMU in agricultural animals and drug-resistant foodborne campylobacteriosis in humans [26]. The study concluded that selection pressure due to AMU in farms can increase the colonisation of animals with a resistant *Campylobacter* spp. but that there was insufficient information to establish a causal relationship between the use of AMs in agricultural animals and the prevalence of resistant foodborne *Campylobacter* in humans.

The European Centre for Disease Prevention and Control (ECDC), with the European Food Safety Authority (EFSA) and the European Medicines Agency (EMA), produced Joint Interagency Antimicrobial Consumption and Resistance Analysis (JIACRA) reports in 2015 and 2017. These reports analyzed the potential relationships between the consumption of AMs and the occurrence of AMR in bacteria isolated from humans and FPAs (the category FPAs included broilers, pigs and cattle for 2013 and broilers, turkeys, pigs and calves for 2014–2015). This integrated analysis was based on the ‘One Health’ approach and considered particular combinations of AMs and foodborne zoonotic bacterial strains (e.g., *Salmonella* spp., *Campylobacter* spp.) considered important to public health, but also commensal indicator bacteria (i.e., *E. coli*, *E. faecalis* and *E. faecium*) [13,14]. In these reports, statistically significant positive associations were observed between the consumption of fluoroquinolones and other quinolones in FPAs and resistance to fluoroquinolones in indicator *E. coli*, *Salmonella* spp., *C. jejuni* and *C. coli* from these animals for the years 2013, 2014 and 2015. Statistically significant associations were also observed between tetracycline consumption in FPAs and resistance to tetracyclines in indicator *E. coli*, *Salmonella* spp. and *C. jejuni* from FPAs for 2013, 2014 and 2015. A statistically significant positive association was observed between macrolide consumption in FPAs and resistance to macrolides in *C. coli* from FPAs for 2014–2015. Chantziaras et al. (2013) assessed correlations between AMU and resistance in commensal *E. coli* isolates from pigs, poultry and cattle in seven European countries (Austria, Belgium, Denmark, Norway, Netherlands, Norway, Sweden and Switzerland). The study showed that the level of use of specific AMs strongly correlates with the level of resistance towards these agents in *E. coli* isolates (e.g., Belgium had the highest level of AMs used and had the highest levels of resistance compared to Norway and Sweden, which had the lowest quantities of AMU and the lowest levels of resistance) [12].

Examples of documented associations between AMU and AMR are shown in Table 1.

### 2.2. Antimicrobial Use in Animals and Antimicrobial Resistance in Humans

The relationship between AMU in animals and AMR in humans has been investigated using a variety of methods. Most studies focused on the transmission pathways of resistant bacteria from animals to humans (i.e., AMR ecology studies). Very few studies investigated the direct effects of usage in animals on the occurrence of resistance in bacteria and its impact on humans. A summary of the findings is described in the following sections.

#### 2.2.1. Molecular Studies Investigating Links between Resistant Bacteria or Genes in Animals and Their Transmission to Humans

Lazarus et al. (2015) conducted a systematic review to investigate whether extra-intestinal *E. coli* infections resistant to expanded spectrum cephalosporins (ESCR-EC) originated from FPAs. Thirty-four studies were identified for inclusion. Six molecular studies supported the transfer of resistance via whole bacterium transmission, which was well characterized among poultry in the Netherlands, but it was not clear if this was a geographic phenomenon or due to limited research in other parts of the world. Thirteen molecular studies supported the notion of transmission of resistance via mobile genetic elements and these studies had a greater diversity of geography and host species of FPAs, thus strengthening the relevance of this observation. Seventeen studies did not support whole bacterium transmission and two did not support mobile genetic elements transmission. Four observational studies supported the hypothesis of zoonotic transmission. The review concluded that a proportion of human ESCR-EC was attributed to FPAs, with poultry being the most likely source, but the quantitative and geographical extent of the problem was not well understood [28].

In a study of multidrug-resistant (MDR) *Salmonella* Typhimurium DT104, whole genome sequencing (WGS) was used to investigate the phylogenetic relationship of the bacterium and its AMR genes through the course of an epidemic [29]. A total of 142 isolates from humans and 120 from animals (70% of which were of bovine origin) in Scotland, obtained between 1990 and 2011, were sequenced; an additional 111 international isolates (from humans and animal sources) were added to the sample. The results showed that the bacterium and its resistance genes were maintained separately within bovine and human populations with limited spill-over in both directions. It was also reported that there was greater diversity of AMR genes in the human isolates compared to the bovine isolates; this indicated that other sources of *S.* Typhimurium DT104 could have contributed to the human resistance, such as other animal reservoirs, imported food, foreign travel and environmental reservoirs. Similar findings were observed in two studies conducted in the United States. The first used genomic analysis to compare livestock–and human-associated *Salmonella* strains from different regions. The results showed that AMR genes were associated with specific hosts, with some overlap between species. These overlaps were observed mainly in *Salmonella enterica* serovar Newport [30]. The second study used WGS to investigate the epidemiology and AMR patterns within *Salmonella* isolates from humans, FPAs and the environment. The results indicated that specific AMR genes were observed in each *Salmonella* serovar [31]. In an attribution study conducted in Denmark, a mathematical model was developed to quantify the contribution of different animal foods and travelling abroad as sources of human infections with resistant *Salmonella* strains [32]. The study found that domestic food was the main source of *Salmonella* infections in humans, but infections with MDR and quinolone-resistant isolates were more commonly caused by imported animal food products and travelling abroad.

Livestock-associated methicillin-resistant *Staphylococcus aureus* (LA-MRSA) clonal complex 398 (CC398) was discovered for the first time in pig farms in the Netherlands and France in the early 2000s [33]. To date, LA-MRSA CC398 has been reported in different livestock species in Europe and worldwide. LA-MRSA is found in the nose or on the skin of livestock without causing clinical signs of infection. Livestock frequently transmit it to humans, with direct livestock contact being the major risk factor for transmission of MRSA CC398. Exposed individuals can become colonized, with LA-MRSA being found asymptomatically in the nose or on the skin. If LA-MRSA enters the body through the skin (e.g., via a wound or a cut), it can cause a local skin infection (e.g., abscess). Occasionally, it can cause invasive diseases such as pneumonia or bloodstream infections (BSIs) [34,35,36]. In Denmark, a study showed that the increasing number of LA-MRSA CC398 BSIs and skin and soft tissue infections, observed between 2010 and 2015, was the result of zoonotic transmission from the expanding pig reservoir [34]. A study of an epidemic of LA-MRSA in Germany was conducted at a hospital located in a region characterized by its high livestock density and considered as a hotspot for the occurrence of LA-MRSA CC398. It showed that CC398 accounted for 9.6% of all local MRSA in 2005 and that this proportion reached 35% in 2013, which increased the burden of MRSA colonization and infection at the hospital [37].

Colistin resistance was mainly transmitted vertically in bacterial clones until the first occurrence of horizontal transfer by a plasmid-mediated colistin resistance gene (mcr-1) in Enterobacteriaceae was first reported in China in 2015 in FPAs and humans [8]. Colistin is a last resort drug in human medicine for systemic treatment of infections caused by MDR *Pseudomonas aeruginosa*, *Acinetobacter baumannii* and Enterobacteriaceae. Following this finding, different countries around the world have reported the presence of these genes or their variants in humans, animals and meat [38,39,40,41,42]. The discovery of this new mechanism of colistin resistance sparked fear because of the potential risk of rapid spread. Although colistin had not been used clinically for a long time because of its toxicity in humans, many countries have used it routinely in farm animals [43]. The mcr-1 gene was found in similar plasmids in the same bacterial species isolated from FPAs, the food of animal origin, humans and the environment, indicating possible transmission between these [43,44,45]. As a risk mitigation policy, EMA recommended a reduction in the use of colistin in animals in the EU, highlighting that this reduction should not be accompanied by an increase in the use of other AMs but should be achieved by combining the reduction with an improvement in biosecurity and farming conditions and vaccination of livestock [43].

The link between animal-derived *E. coli* strains and urinary tract infections (UTI) in humans was investigated in a study in Denmark. A sample collection of *E. coli* strains from cattle, pigs, poultry and meat products from these animals was compared to a collection of *E. coli* isolates from healthy humans and those with a UTI. These strains had been identified previously as exhibiting virulence genotypes. A comparison of virulence genes, phylotypes, pulse-field gel electrophoresis and antimicrobial susceptibility testing (AST) demonstrated a clonal link between *E. coli* from animals, meat and humans. This led to the conclusion that *E. coli* UTI in humans could be the result of zoonotic transmission [20].

#### 2.2.2. Presence of AMR Bacteria in Food Products of Animal Origin

Antimicrobial resistant bacteria are present in the food chain; this constitutes a potential route for human exposure to AMR bacteria or resistance genes. This can occur due to the contamination of food during agricultural production, or the presence, addition or cross-contamination with resistance genes and resistant bacteria during food processing [46].

Two systematic reviews were conducted between 1999 and 2016 in the UK and Switzerland to investigate the occurrence of AMR in bacteria present in retail food [47,48]. The UK review looked at studies from the UK and countries exporting to the UK. It showed that there was a lack of data on British-produced food, and to a lesser extent, on countries exporting to the UK, except for northern European countries. For poultry meat in the UK, an increasing trend of fluoroquinolone resistance in *Campylobacter jejuni* isolates had been observed since 2001. Resistance levels to ciprofloxacin and nalidixic acid in 2001 were 12.6% and 15.6%, respectively. These levels increased to 21.7% and 23.7% in 2005, and were up to 50% and 51% in 2014–2015 for ciprofloxacin and nalidixic acid. For exporting countries to the UK, high levels of resistance to ciprofloxacin and nalidixic acid in *C. jejuni* for poultry meat were also observed in studies from the Netherlands (63.4% in 2014) and Poland (up to 100%). For Denmark, an increase in ampicillin resistance in bacteria isolated from pork was observed in *Salmonella* isolates (up to 73% in 2013) and in *E. coli* isolates (up to 33% in 2012) [47].

The review produced in Switzerland [48] targeted studies linked to Switzerland and the Swiss retail sector, i.e., studies from Switzerland as well as countries exporting food to Switzerland. The largest number of AMR-positive samples was observed in raw meat products; this was partly explained by an overrepresentation of studies on raw meat (of the 313 studies included in the review, a total of 160 studies contained data on the testing of raw meat). Major resistance levels among Gram-negative foodborne pathogens were observed in *Campylobacter* spp. against fluoroquinolones (33% of the isolates) and tetracyclines (25% of the isolates). In *S.* Typhimurium, 20% of the isolates were resistant to aminoglycosides, 20% resistant to penicillins and 18% resistant to tetracyclines. For *S. enteritidis*, the percentage of isolates resistant to aminoglycosides and fuoroquinolones were 24% and 23%, respectively [48].

In the latest EU summary report on AMR in zoonotic and indicator bacteria from humans, animals and food in 2017 [38], high levels of resistance were reported in *Salmonella* isolates from pigs’ carcasses to ampicillin, sulfamethoxazole and tetracycline (53%, 59.5% and 56.8%, respectively). Countries showing high levels of resistance to these AMs were Spain, Ireland, Germany, Belgium, France and Denmark. For fluoroquinolone, resistance was detected at moderate levels in member states (only countries submitting data for more than 10 isolates were considered) from southern and northern Europe (16.7% in Spain, 12% in Croatia, 10.5% in Italy and 13% in Ireland) while low levels were detected from Western and Eastern Europe (7.1% in Belgium, 6.5% in Germany, 4.8% in the Czech Republic and 4.2% in Poland); Portugal showed low levels (2.9%), and no resistance to ciprofloxacin was detected in other member states. Multidrug resistance in *Salmonella* spp. from pig carcasses was also high (47.4%).

Provided that the DNA is not digested or destroyed through processing, AMR genes in microbes in food can spread to other bacteria in the human gut [4]. The public health relevance of such a gene transfer has yet to be quantified. Good hygiene and manufacturing practices, including cleaning, chilling, and avoiding cross-contamination, reduce the exposure to bacteria, including those carrying AMR genes. In addition, sufficient cooking is crucial as it destroys bacteria present in food [49].

#### 2.2.3. Association between Antimicrobial Use in Food Animals and Resistant Bacteria in Humans

A study from Canada showed a positive correlation between ceftiofur-resistant *Salmonella enterica* serovar Heidelberg, isolated from retail chickens, and incidence of ceftiofur-resistant *S*. Heidelberg infections in humans across Canada. After the voluntary withdrawal of ceftiofur use in hatcheries in Canada in 2005, a decrease in ceftiofur-resistant *S*. Heidelberg in chickens (62% to 7%) and humans (from 36% to 8%) was observed, followed by an increase in resistance levels in both species (from 7% to 18% in chicken and from 8% to 12% in humans) after the reintroduction of its use in young chicks to control omphalitis in 2007 [27]. This is a rare example of a direct temporal association between AMU in animals and AMR in humans. In the US, enrofloxacin was withdrawn from use in poultry in 2005 after a rapid increase in human infections with fluoroquinolone-resistant *Campylobacter* species following the licensing of two fluoroquinolones for poultry in 1995 and 1996 (none of 297 human campylobacter isolates were resistant to ciprofloxacin in a survey conducted in 1990 by the Centers for Disease Prevention and Control; this level increased to 18% in 1999 based on the data collected by National Antimicrobial Resistance Monitoring system) [50].

In the systematic review conducted by Tang et al. (2017), evidence of the effect of restricting AMUs in FPAs on humans was limited and less robust; the analysis of 13 studies showed a reduction of 24% in AMR bacteria in humans due to the reduction in AMU in animals. This impact was mainly observed in people directly in contact with livestock, though source attribution to the food chain was not possible. The authors acknowledged the limitations of the review and the high level of heterogeneity observed between the studies. They concluded that these results cannot be extrapolated to the human population as a whole because of the limited number of studies available [51].

In the 2017 JIACRA report, multivariate analyses showed that resistance to fluroquinolones and other quinolones in *Salmonella* spp. and *Campylobacter jejuni* from humans was significantly associated with resistance bacteria from FPAs, which in turn was significantly linked to the consumption of fluoroquinolones and other quinolones from such animals. A significant association was also observed between the use of macrolides in animals and resistance to *Campylobacter coli* in humans [14].

The impact of restricting antibiotic usage in FPAs on resistant bacteria in humans has been investigated using a mathematical model in a recent study [52]. The objective of this study was to better understand the dynamics of the relationship between antibiotic resistance in humans and animals, and to identify the model parameters that have the greatest impact on the model results, i.e., the reduction in transmission risk. The results showed that reducing the amount of antibiotic usage in FPAs had little impact on resistant bacteria in humans if used in isolation, and that reducing the rate of transmission of resistance from animals to humans may be more effective.

#### 2.2.4. Studies with Limited Evidence of an Association between AMR in Humans and Food-Producing Animals

In Denmark, CTX-M-15 is the dominant resistance genotype for Extended Spectrum Beta-Lactamases (ESBLs) in *E. coli* and *Klebsiella* spp. in humans (around 50–70% of all ESBLs). Yet, this gene has rarely been found in Danish production animals, indicating that there is little transfer of these strains from livestock to humans in Denmark (e.g., in pigs and pork, the most common ESBL type was CTX-M-1) [20]. A similar result was found in a study conducted in Sweden to investigate food as a potential source and dissemination of ESBL-producing *E. coli* to humans (ESBL-producing Enterobacteriaceae including *E. coli* is the most commonly reported resistance type in Sweden). The study analyzed data from approximately 5300 samples taken from foods (domestic and imported), farm animals, healthy volunteers, severely ill patients, the environment and sewage water. The comparison of the genes encoding ESBL showed that there are three separate populations of genes encoding ESBL in Sweden, one in Swedish foods and farm animals, one in imported foods, and one in humans and the environment. The results indicated that food made a limited contribution to the occurrence of ESBL-producing *E. coli* within the healthcare sector in Sweden, though the exact level of contribution was not reported [53].

The prevalence and types of ESBL-producing *E. coli* in raw retail beef, chicken, pork, fruit and vegetables from five UK regions in 2013–2014 were investigated. The results demonstrated that 2% of beef and pork and about 65% of chicken were positive to CTX-M type ESBL producing *E. coli* but all of the fruit and vegetables tested negative. None of the ESBLs found included CTX-M-15, which is the predominant human clinical isolate in the UK [54]. Another study in the UK used genomic surveillance to investigate the role of livestock as a reservoir for drug-resistant *E. coli* that infect humans [55]. A total of 431 *E. coli* isolates (including 155 ESBL-producing isolates) from livestock farms (cattle, pig and poultry) and meat in the East of England were compared to isolates from 1517 patients with BSIs in the UK. The study concluded that there was limited evidence that AMR pathogens causing severe *E. coli* human infection originated from livestock in that region.

To quantify molecular similarities of ESBL/AmpC-producing *E. coli* (ESBL/AmpC-EC) from humans, animals, food and the environment, pooled data on ESBL/AmpC-EC isolates were recovered from 35 studies in the Netherlands comprising more than 27,000 samples [56]. The results showed close gene similarity between human farming communities and their animals (broilers and pigs). Isolates from people in the general population also had higher similarities to those from human clinical settings, surface and sewage water and wild birds, while similarities to livestock or food reservoirs were lower. This suggests that livestock reservoirs are not major contributors to ESBL/AmpC in humans. Two exposure assessment studies have been conducted to estimate the relative contribution of different types of meat to the exposure of consumers to ESBL/AmpC-EC. The first one was conducted in Denmark and the results showed that broiler meat represented the largest part (83.8%) of the estimated ESBL/AmpC contaminated meat compared to pork (12.5%) and beef (3.7%) [57]. The genotype with the highest relative contribution to humans (CMY-2) was rarely found in human infection in Denmark, which suggests that meat might constitute a less important source of ESBL/AmpC exposure to humans in Denmark. The second study was conducted in the Netherlands and found that the consumption of beef products led to a higher exposure than chicken products, although the prevalence of ESBL/AmpC on raw chicken meat was much higher than on beef [58]. The relative risk to public health of this exposure is yet to be investigated.

Gouliouris et al. (2018) investigated the relationship between *Enterococcus faecium* strains in humans and livestock. Six hundred *E. faecium* isolates (including Vancomycin-resistant) from livestock farms (cattle, pig and poultry), retail meat, and wastewater treatment plants in the UK were compared to almost 800 genomes of *E. faecium* isolates from patients with BSIs in the UK and Ireland. The findings showed that the majority of *E. faecium* strains infecting patients were distinct from those from livestock, with limited sharing of strains and resistance genes [59].

Another example relates to the resistance to carbapenems that is emerging in humans and constitutes a public health concern. Carbapenems are classified by the WHO as critically important AMs because they are used for the treatment of serious infections in humans and are considered the last line therapy for infections caused by MDR Gram-negative bacteria [23]. Although this class of AM is not authorized for use in animals, carbapenem resistance in bacteria from animals has been reported in very few cases, indicating that dissemination from humans to animals, directly or through environmental routes, may occur [13].

Examples of resistance genes that are of importance to human health are presented in Table 2 (non-exhaustive list).

### 2.3. Antimicrobial Resistance in Bacteria Linked to Food Processing Practices

The use of disinfectants in food processing premises is important in decreasing the risk of contaminated food products reaching consumers. There have been concerns that bacteria exposed to disinfectants could develop resistance to these, and consequently have a higher risk of developing AMR [60]. In order to examine the prevalence of biocide-resistant *Salmonella* spp. and to assess if there was a correlation between susceptibilities to biocides and AMs, and the impact of cleaning and disinfection on the selection of isolates with changed susceptibility, Gantzhorn et al. (2014) conducted a study in six Danish pig slaughterhouses. The susceptibility toward three different biocides, triclosan and two commercial disinfection products, Desinfect Maxi (a quaternary ammonium compound) and Incimaxx DES (an acetic compound), was determined. The study found no resistance towards the biocides tested but found that isolates obtained after cleaning and disinfection had an increased resistance toward one of the disinfectants (Incimaxx DES) compared to isolates obtained before cleaning and disinfection. This indicated the possibility of the selection of strains that were more tolerant to biocides due to cleaning and disinfection. Also, a weak correlation was observed between susceptibility to biocides and some AMs; for example, a negative correlation between triclosan and polymyxin B, and a positive correlation between Desinfect Maxi and tobramycin. The study concluded that no evidence was found of decreased susceptibility toward the three biocides tested among isolates obtained from Danish slaughterhouses.

Other studies conducted in laboratories found correlations between exposure to biocides and increased resistance to AMs. Alonso-Hernando et al. (2009) tested *Salmonella enterica* and *Listeria monocytogenes* strains against sub-inhibitory concentrations of decontaminants (trisodium phosphate, acidified sodium chlorite (ASC), citric acid (CA), chlorine dioxide (CD) or peroxyacetic acid (PA)) applied in poultry processing. The AMR patterns were compared before and after exposure, and an increase in resistance to various AMs after exposure to chemicals was observed (e.g., *S. enterica* serotype Typhimurium was sensitive to streptomycin and became resistant after exposure to ASC, CD and PA) [61]. Condell et al. (2012) investigated the tolerance of a collection of susceptible and MDR *Salmonella enterica* strains to seven food-grade biocide formulations and explored their ability to adapt. The results showed that after exposure, a high level of tolerance was selected for a number of *Salmonella* serotypes and that most tolerant isolates displayed changes in their patterns of susceptibility to AMs [62].

In the United States, the use of triclosan in antibacterial soaps was banned because of concerns regarding the health effects of long-term use and because of the concerns that it can lead to AMR [40]. Biocides such as triclosan, unlike AMs, exert their antibacterial activity using non-specific targets in the cells and the bacteria uses its natural defense mechanisms to toxic compounds that are the drug efflux pumps. These pumps are the main contributors to MDR because, once they are expressed, they will not only transport biocides but also AMs, antiseptics, heavy metals and dye [40].

To investigate the impact of food processing on the transfer of AMR bacteria to humans, a review was conducted by Verraes et al. (2013). The results showed that the effects of food processing (such as heat treatment, cooling, acidification, modified atmosphere packaging, freezing, mild pasteurization and ultraviolet radiation treatment) on bacteria were variable, but in general there was a decrease in the number of bacteria when these techniques were applied appropriately. Dead bacteria are not able to perform conjugation, and heat treatment that kills bacteria reduces the risk of AMR gene transfer. Raw food was considered to pose the highest risk to consumers because resistant bacteria are not killed by any treatment. This study also showed that minimal processing or preservation treatments that cause sublethal stresses (damage but not kill bacteria, or slow but not stop bacterial growth) to bacteria can trigger several mechanisms in bacterial cells which could increase the probability of AMR genes transfer. Stress in bacteria can cause changes in the cells that may impact AM susceptibility through expression of resistance genes [63]. However, there is still a lack of information about the AMR risk associated with use of these techniques. It was also reported that microorganisms intentionally added to foodstuffs, such as starter cultures (e.g., *Lactobacillus*, *Lactococcus, Enterococcus* spp.), probiotics, and biopreserving microorganisms, may contain AMR genes and may transfer these to gut bacteria [46].

Jans et al. (2018) also confirmed the presence of AMR bacteria in fermented products, particularly for *Enterococcus* spp., and starter cultures, with resistance reported against aminoglycosides, fluoroquinolones, glycopeptides, penicillins and tetracyclines. The authors were not able to quantify the risk from these types of exposure because of the limited availability of data and concluded that systematic surveillance needs to be applied to collect the data needed to assess the public health risk.

## 3. Discussion

In this review, we covered comprehensively and in an integrated manner the links between the use of antimicrobials in the food chain and the occurrence of AMR in people and animals. Previous reviews focused mostly on distinct aspects in animal food chains, or human and animal populations. For example, the two systematic literature reviews conducted in the UK and Switzerland [47,48] assessed AMR bacteria occurrence in retail food and the review conducted by Verraes et al. (2013) investigated the impact of food processing on the transfer of AMR to humans. Other reviews focused on one type of bacteria, such as MRSA or *Campylobacter* [26,35]. Hence, this review is an addition to, and complements, previous work.

Correlations between AMU in animals and the occurrence of AMR have been demonstrated in various studies. Also, interventions to reduce the use of AMs in animals were effective in reducing AMR in these animals [16,18,20,64]. The benefits of reduction in AMU in animals on AMR in humans were difficult to quantify, with an association reported mainly for people in contact with FPAs [51]. This may be explained by the complexity of AMR and the contribution of factors other than the quantity of AMs used in animals on AMR in humans, such as parallel AMU in humans both in hospitals and at community level, exposure to resistant bacteria present in the environment and in fresh food products and the use of AMs in companion animals with which humans are in close contact [1,65,66]. Also, different bacteria utilize different genetic resistance mechanisms and are transmitted by different pathways [4]. Occupational exposure of farm workers and veterinarians has been reported, and this is particularly relevant in the case of LA-MRSA where high levels of nasal carriage were detected in farmers and veterinarians. Indirect livestock exposure can also constitute a source of colonization, such as in households and farm visits [37,38].

Resistant bacteria are present in the food chain, which constitutes an exposure route for humans, and good hygiene practices need to be applied to reduce this risk [47,48]. The impact of food processing and preservation techniques is variable but, in general, the number of bacteria is reduced when these techniques are applied, with raw food presenting the highest risk because it is not subject to any treatment. Food processes that reduce the load of or kill bacteria decrease the risk of transmission of AMR. In terms of the health consequences for consumers exposed to resistance genes in foods, current evidence suggests that the health impact of the presence of resistance genes in processed foods is likely to be limited. There remains uncertainty regarding the biological consequences of ingested resistance genes in bacteria that are able to survive digestion, such as gastrointestinal pathogens, and also the risk due to AMR genes transfer (e.g., from commensals originating from animal reservoirs to human pathogens). Commensals can constitute a significant reservoir of AMR and it is very important to understand the dynamics of the transfer of resistance genes between commensals and pathogen bacteria to be able to quantify its contribution towards this transfer in order to be able to assess its public health impact. The risk of consumer exposure to AMR depends also on food preparation and consumer preference. Although most raw meat products are cooked before consumption, which decreases the bacteria quantity and reduces the risk of AMR, the increasing demand for raw (e.g., Sushi) and undercooked food (e.g., rare burgers) may result in an increased risk of exposure to AMR [46,48].

Measures to reduce the use of AMs as far as possible (e.g., campaigns for responsible use) should remain a priority to reduce the pool of resistant microorganisms circulating in the animal population. Good husbandry practices also need to be applied to reduce the colonization of animals with resistant pathogens and therefore reduce the need to use AMs and the risk of transmission of resistant bacteria in the food chain. This was also emphasized in a joint scientific opinion by EMA and EFSA on measures to reduce the need to use AM agents in animal husbandry in the EU and the resulting impacts on food safety [67]. The report concluded that it was difficult to assess the impact, but that overall it was ‘reasonable to assume’ that a reduction in AMU would result in a reduction in AMR in bacteria from FPAs and food. Importantly, reducing resistant bacteria in FPAs is not only necessary because of potential transmission risks to humans, but also to safeguard the health and welfare of animal populations and thereby secure the production of animal-sourced foods and contribute to food security. Data showing the impact of a reduction in AMU on resistance were mainly available from countries that had pursued integrated surveillance strategies for a long time. Hence, without long-term monitoring, changes in AMU and AMR, as well as the effects of specific interventions, are difficult to capture. Although some studies reported statistically significant associations rather than causality, those findings are important because they can be used to generate hypotheses and design studies to test these hypotheses [12,13,14].

Current monitoring of AMR is based on phenotypic susceptibility testing but many surveillance systems started incorporating WGS to improve the diagnosis and control of infectious diseases [68]. Whole genome sequencing and metagenomics constitute an important advance in technology, allowing better understanding of AMR ecology and it may be expected that more information will be available soon that will guide the planning of more targeted investigations of the different associations identified and the implementation of better evidence-based interventions. Several studies in this paper used molecular techniques to investigate associations between resistance genes in humans, animals, food and the environment (e.g., colistin resistance, *Salmonella*, LA-MRSA, ESBLs), and new evidence is emerging which will enable a better understanding of the links between resistance in the different populations [29,30,31,55,56,59]. However, because of the complex transmission routes and the different reservoirs of AMR genes, these studies provide information about a part of the pathway and not the entire pathway from ‘farm to fork’. The application of source attribution approaches to AMR pathogens or to AMR genes is recognized as an important tool to identify the most important sources and transmission routes for human exposure to AMR, which is crucial for the prioritization of risk management along the food chain [69]. There are few published examples of source attribution studies [32,57,58] and more studies are needed that would address the gaps in knowledge and the weaknesses of the existing studies. These studies need to use a variety of methods and include experts from different disciplines, such us microbiologists, veterinarians, doctors, genetic specialists, epidemiologists and social scientists, to be able to capture the different factors involved in the use of AMs in animal production that could pose a risk to humans. Some of the limitations of the use of these methods are the restricted availability of AMR surveillance data from animals, food, human and the environment, and scarcity of data on gene transfer and spread of AMR genes.

None of the literature identified gave conclusive results about the exact role that the food chain plays in AMR in humans, and significant evidence gaps still exist. These include the proportion of AMR development in humans attributable to the food chain, better understanding of resistance genes transfer between bacteria including non-pathogenic ones (commensals) and the impact of reducing AMR in FPAs on AMR in humans in contact with these animals and on consumers of food. The development of integrated surveillance systems for AMR and AMU with harmonized designs across human and animal populations, with linkages to samples from food and the environment, is essential for a better understanding and management of AMR. The development and spread of AMR in the environment are also of increasing concern and there is a lack of data on the role of environmental factors in the transmission of resistance. Antimicrobials can contaminate the environment through animal waste, human waste and manufacturing waste. This can provoke the development of resistance in bacteria present in soil, crops and water sources, and, therefore, may constitute a significant pathway for the transmission of AMR to humans and animals [1]. A holistic approach is needed to better understand the microbiome (the collective genomes of microorganisms inhabiting a particular ecosystem) associated with the food chain and the environment and the composition of the resistome (genes encoding resistance to AMs); this will help to explain the role of the food chain in AMR spread and emergence in a population.

This review has limitations, for example, some studies investigating the links between AMR in specific bacteria in the food chain and AMR in humans may have not been picked up because of the search strings used and also the quality of the studies was not formally assessed. On the other hand, the inclusion of different studies including systematic reviews, primary studies and surveillance reports has enabled provision of an overview of a very wide but important aspect of understanding AMR. In addition, this study enables a discussion of the published evidence and offers recommendations for future research and implications for policy.

## 4. Methods

The scientific literature contained in PubMed and Science Direct were searched using a title and abstract. Google searches were used to identify relevant grey literature. The search terms used were: ‘antimicrobial resistance’, ‘antibiotic resistance’, ‘antimicrobial use’, ‘antibiotic use’, ‘food chain’, ‘food producing animals’, ‘agriculture’ and ‘livestock’. Apart from the general search in the databases mentioned above, a targeted search was performed to identify the grey literature in the Netherlands, Denmark and Sweden, where integrated surveillance of AMU and AMR in humans, animals and food has been conducted for several years, and in European surveillance reports. No restrictions about publication type or year were applied. The search was conducted in May 2017 and then updated in April 2019. The only terrestrial animals that were considered were poultry, sheep, goats, cattle, calves, pigs. The following exclusion criteria were applied: (1) the study did not assess an association between AMU and AMR; (2) the study referred to animals other than terrestrial animals (e.g., wildlife, companion animals); (3) the study was in a language other than English. The references were reviewed and exemplary studies examining the links between AMU and AMR in the food chain and AMR in humans were selected; these included systematic reviews, primary studies and surveillance reports. Studies describing the impact of interventions to reduce AMU in animals on AMR in animals and humans were also included because they were considered as presenting an indirect way to demonstrate the evidence of the links investigated. Antimicrobial resistance is used throughout this document to include resistance to antibacterial, antiviral and antiparasitic agents, although the focus is primarily on bacterial resistance to antibacterial agents.

## 5. Conclusions

Although the extent to which the food chain contributes to AMR is difficult to quantify due to its complex epidemiology, there is some evidence of links between AMU in the food chain and AMR in humans. Until better evidence become available, the prudent use of AMs should remain a priority for both human and animal populations to preserve the efficacy of AMs and reduce the pool of resistance. This needs to be supported by other strategies to reduce AMR, including continuing to educate health professionals to help them with their treatment decisions, public awareness campaigns designed to help reduce inappropriate patient and client expectations related to antibiotic prescribing, improvement of hygiene in healthcare settings and the food chain, improvement of vaccination uptake for preventable diseases, and the development of new antimicrobial drugs and rapid diagnostic tests. A One Health collaboration across sectors and disciplines on AMR will enhance our knowledge of AMR emergence and transmission and will enable us to implement more evidence-based interventions to manage the risk of AMR resulting from the food chain; these need to be accompanied by continuous monitoring to document the effects of interventions and learn from them.

## Figures and Tables

**Table 1:** Examples of associations between the use of some antimicrobials and resistance to these antimicrobials in specific bacteria.

Antimicrobials	Hosts	Associated Bacteria	Reference
Cefotaxime (a 3rd generation cephalosporin)	Broilers	*E. coli*	[16]
Cephalosporin	Pigs	*E. coli*	[21]
Avoparcin (a glycopeptide)	Broilers	*E. faecium*	[24,25]
Tylosin (a macrolide)	Pigs	*E. faecium* and *E. faecalis*	[25]
Tylosin (a macrolide)	Broilers	*E. faecium*	[25]
Virginiamycin (a streptogramin)	Broilers	*E. faecium*	[25]
Avilamycin (an oligosaccharide)	Broilers	*E. faecium*	[25]
Ampicillin	Livestock	*E. coli*	[22]
Fluoroquinolones and other quinolones	Food producing animals	*E. coli*, *Salmonella* spp., *Campylobacter jejuni* and *Campylobacter coli*	[13,14]
Tetracyclines	Food producing animals	*E. coli*, *Salmonella* spp. and *Campylobacter jejuni*	[13,14]
Macrolides	Food producing animals	*Campylobacter coli*	[13,14]
Ceftiofur (a 3rd generation cephalosporin)	Chickens	*E. coli* and *Salmonella enterica* serovar Heidelberg	[27]

**Table 2:** Examples of bacterial genes and their roles in antimicrobial resistance.

Bacterial Resistance Genes	Role in Antimicrobial Resistance
Genes encoding for extended-spectrum beta-lactamase (ESBL-) and AmpC beta-lactamase (AmpC-) in Enterobacteriaceae (for ex. CTX-M type)	Exhibit resistance to a wide range of β-lactam antibiotics, including penicillins and 3rd and 4th generation cephalosporins in Gram negative bacteria. They are one of the fastest emerging resistance problems in both humans, companion and production animals worldwide. Third and 4th generation cephalosporins are now classified as Highest Priority Critically Important Antimicrobials (HPCIAs) by WHO.
Carbapenemase-encoding genes in Enterobacteriaceae	Carbapenems are classified by WHO as HPCIAs. Resistance to carbapenems is emerging in humans and constitute a major public health concern. Although carbapenems are not licensed for use in animals, carbapenem resistance has been isolated in bacteria from companion, food-producing animals and wildlife.
Livestock-associated methicillin-resistant *Staphylococcus aureus* (LA-MRSA) Clonal Complex 3398	Found in the skin and nose of pigs, cattle and horses without causing a disease and they can transmit it to humans. Exposed individuals can become colonised asymptomatically; LA-MRSA can cause skin infections and occasionally invasive disease (septicaemia). Veterinarians and farmers are at high risk of exposure through their occupations.
Plasmid mediated colistin resistance (mcr genes)	Colistin (Polymyxin E) is a last resort antibiotic in humans. This new gene is transmitted horizontally, which can cause a higher risk of spread between animals and humans. Colistin resistance has been identify in livestock (pigs and poultry) and animal-derived food and in humans.
Vancomycin resistance encoding genes in *Enterococcus faecium*	A major cause of nosocomial infection and is categorized as high priority by the WHO global priority list of antibiotic-resistant bacteria. Vancomycin-resistant *Enterococci* (VRE) have been isolated from food-producing animals as a result of the previous use of avoparcin as a growth promoter (Note: antibiotic growth promoters have been banned in Europe since January 2006 but are still used in other countries).

## Supplementary Materials

The following are available online at https://www.mdpi.com/2079-6382/9/2/49/s1, Table S1: Summary of studies investigating the links between antimicrobial use in the food chain and antimicrobial resistance in humans and animals.
