# Supplementary material for: Overview of Evidence of Antimicrobial Use and Antimicrobial Resistance in the Food Chain
Source: Antibiotics (Basel). 2020 Jan 28;9(2):49. doi: 10.3390/antibiotics9020049 (PMC7168130; doi:10.3390/antibiotics9020049)
Supplement: Supplementary file 1 [file antibiotics-09-00049-s001.pdf]

antibiotics

Table S1. Summary of studies investigating the links between antimicrobial use (AMU) in the food chain and antimicrobial resistance (AMR) in humans and animals.

Reference	Country	Description of the study	Main findings
Antimicrobial use and antimicrobial resistance in animals			
[1]	Denmark	Investigation of the effect of the termination of the use of glycopeptides, macrolides, oligosaccharides, and streptogramins as growth promoters on the occurrence of resistance in fecal enterococci from food animals (*E. faecium* and *E. faecalis* isolates from pigs, and *E. faecium* isolates from broilers).	-Resistance to erythromycin (a macrolide) among *E. faecium* and *E. faecalis* isolates from pigs was almost 90% between 1995 and 1997 and these levels decreased to 46.7% and 28.1% for *E. faecium* and E. faecalis respectively following the decrease in tylosin use. Erythromycin resistance among *E. faecium* from broilers decreased from 76.3% in 1997 to 12.7% in 2000.<br>-Resistance to vancomycin (a glycopeptide) among *E. faecium* in broilers decreased from 72.7% in 1995 to 5.8% in 2000 following a ban of avoparcin (a glycopeptide) in 1995.<br>-The use of virginiamycin increased from 1995 to 1997 and was followed by an increase in virginiamycin resistance among *E. faecium* isolates in broilers from 27.3% to 66.2%. Following the ban of its use in 1998, virginiamycin resistance decreased to 33.9% in 2000.<br>-The use of avilamycin (an oligosaccharide) increased from 1995 to 1996 followed by an increase in avilamycin resistance among *E. faecium* isolates from broilers, from 63.6% to 77.4%. Avilamycin use decreased since 1996 and this was followed by a decrease in resistance to 4.8% in 2000.
[2]	Denmark	The paper presents selected examples and experiences from the first 11 years of monitoring and reporting of antimicrobial (AM) consumption and AMR from animals, food, and humans as a part of the Danish Integrated Antimicrobial Resistance Monitoring and Research Programme (DANMAP)	Monitoring of AMR and AMU and a range of research activities related to DANMAP have contributed to restrictions or bans of use of some AMs in food animals in Denmark and other European Union countries.
[3]	Denmark	Investigation of the effect of a voluntary ban on cephalosporin usage in the Danish pig production on the prevalence of extended-spectrum cephalosporinase (ESC)-producing *E. coli* in pigs and pork	Following the ban, the occurrence of ESC-producing *E. coli* declined in pigs at slaughter from 11.8% in 2010 to 3.6% in 2011, and from 11% in 2010 to 0% in 2011 in pig farms.
[4]	Seven European Countries	Study assessing correlations between AMU and resistance in commensal *E. coli* isolates from pigs, poultry and cattle in seven European countries	The level of use of specific AMs strongly correlates to the level of resistance towards these agents in commensal *E.coli* isolates in pigs, poultry and cattle.

		(Austria, Belgium, Denmark, Norway, Netherlands, Norway, Sweden and Switzerland)	
[5]	Denmark	Danish Integrated Antimicrobial Resistance Monitoring and Research Programme.	Presents the results of monitoring the antimicrobial use and antimicrobial resistance in food animals, food and humans in 2015
[6]	The Netherlands	Quantitative assessment of the association between the use of AMs in animals and resistance levels in commensal indicator *E. coli* over a period that saw a major reduction in AMU within the four major livestock production sectors (broilers, pigs, veal calves and dairy cattle)	The policy implemented to reduce AMU had an impact in decreasing *E. coli* resistance, with the most robust associations being in pigs and veal calves. Resistance to widely used AMs (e.g. penicillins, tetracyclines) was, in relative terms, less influenced by drug use changes over time than resistance to newer or less prescribed AMs (e.g. third-/fourth-generation cephalosporins, fluoroquinolones).
[7]	United States, Canada and Denmark	A systematic literature review (from 2010 to 2014) to examine the evidence of a relationship between AMU in agricultural animals and drug-resistant foodborne campylobacteriosis in humans.	There is evidence that on farm antibiotic selection pressure can increase colonization of animals with drug-resistant *Campylobacter* spp., however a causal relationship between use of antimicrobials in agricultural animals and prevalence of drug-resistant foodborne campylobacteriosis in humans could not be established.
[8,9]	European Union	Joint reports on the integrated analysis of the consumption of AMs and occurrence of AMR in bacteria from humans and food-producing animals. The data originate from five different EU-wide surveillance systems run ECDC, EFSA and EMA.	The reports present an analysis of the potential relationships between the consumption of AMs and the occurrence of resistance in bacteria isolated from humans and food producing animals
[10]	The Netherlands	Monitoring of Antimicrobial Resistance and Antibiotic Usage in Animals in 2016	Presents the results of AMU data in food animals and resistance data in bacteria of animal origin.
[11]	Belgium	Investigation of an association between antibiotic use and resistance with a focus on commensal *E. coli* from livestock (veal calves, young beef cattle, pigs and broiler chickens) between 2011 and 2015	AMU decreased by 15.9% between 2011 and 2015, except for florfenicols. Positive correlations were found between resistance and the use of the corresponding antimicrobial class during the study period for most AMs (significant for ampicillin and borderline significant for colistin, sulfamethoxazole, trimethoprim and tetracycline). For chloramphenicol and gentamicin, the correlation was negative. Positive correlations were found between total AMU and resistance (significant for ampicillin and borderline significant for ciprofloxacin, nalidixic acid, sulfamethoxazole, tetracycline and trimethoprim)
Molecular studies investigating links between resistant bacteria or genes in animals and their transmission to humans			
[12]	France	Molecular study, using multilocus	The results suggest that the high risk for

		sequence typing, to describe the characteristics of nasal *S. aureus* strains from farmers and controls and the relationships between strains, and investigate their possible animal origin by comparing them with strains isolated from infected pigs from the same geographic area.	nasal *S. aureus* colonization that was reported in pig farmers was due to strains exchanged with swine; 25 (57%) of the 44 pig farmer isolates grouped together with the swine isolates.
[13]	Denmark	Source attribution study to quantify the contribution of each major animal-food sources to human salmonellosis caused by antimicrobial resistant bacteria	Domestic food products accounted for 53.1% of all cases, mainly caused by table eggs (37.6%). 19% of cases were travel related and 18% could not be associated with any source. Imported food products accounted for 9.5% of all cases with the most important source being imported chicken.
[14]	Scotland	Genomic study using whole genome sequencing to investigate the phylogenetic relationship of *Salmonella* Typhimurium DT104 and its AMR genes from humans and livestock (70% of which were of bovine origin) through the course of an epidemic.	The results showed that the bacterium and its resistance genes were maintained separately within human and animal populations with limited spill-over in both directions. It was also reported that there was greater diversity of AMR genes in the human isolates compared to the bovine isolates; this indicated that other sources of S. Typhimurium DT104 could have contributed to the human resistance such as imported food, foreign travel, and environmental reservoirs
[5]	Denmark	Described previously	Described previously
[15]	Denmark	Investigation of the presence of the plasmid-mediated colistin resistance gene mcr-1 in *E. coli* isolates from food animals, food and human bloodstream infections	Mcr- 1 was detected in an *E. coli* isolate from a Danish patient with bloodstream infection and in five *E. coli* isolates from imported chicken meat.
[16]	Not applicable	A systematic review to investigate if food-producing animals are a source of extra-intestinal expanded-spectrum cephalosporin-resistant *E. coli* (ESCR-EC) infections in humans	There is evidence that a proportion of human extra-intestinal ESCR-EC infections originates from food producing animals. Poultry, in particular, is probably a source, but the quantitative and geographical extent of the problem is unclear and requires further investigation
[17]	Great Britain	Characterization using whole genome sequencing of three colistin-resistant isolates (two *E. coli* and one *Salmonella* Typhimurium) detected in a pig farm	Identification of mcr-1 gene in Enterobacteriaceae which confirms its presence in livestock in Great Britain
[18]	European Union (EU)	An updated advice on the use of colistin products in animals within the EU prepared by the EMA following a request from the European Commission	As a risk mitigation policy, EMA recommended the reduction on the use of colistin in animals in the EU.
[19]	China	Description of the emergence of the first plasmid-mediated polymyxin resistance mechanism, mcr-1, in Enterobacteriaceae in China. In	Polymyxin resistance was shown to be due to the plasmid mediated mcr-1 gene. *E. coli* carrying mcr-1 was found in 78 (15%) of 523 samples of raw meat and

		addition, the prevalence of mcr-1 was investigated in *E. coli* and *Klebsiella pneumoniae* strains collected from five provinces between April 2011 and November 2014.	166 (21%) of 804 animals during 2011–2014, and 16 (1%) of 1322 samples from inpatients with infection.
[20]	United States	Genomic study using whole genome sequencing to compare AMR *Salmonella enterica* serovars Typhimurium, Newport, and Dublin isolated from dairy cattle and humans at the genotypic and phenotypic levels.	The results showed that while many AMR genes and phenotypes were confined to human isolates, overlaps between the resistomes of bovine and human-associated *Salmonella* isolates were observed on numerous occasions, particularly for *S. Newport*.
[21]	Great Britain	Investigation of archived pig material originating in Great Britain from 2013 to 2015 to determine the occurrence of mcr-1-harbouring *E. coli* and characterize mcr-1 plasmids	Mcr-1 was detected on two pig farms through surveillance of caecal samples collected in 2015 from pigs at slaughter. Mcr-1 were also detected in isolates from two porcine veterinary diagnostic submissions in 2015. The low number identified suggests that mcr-1 is uncommon in *E. coli* from pigs within GB. High sequence similarity was found between mcr-1 plasmid draft genomes identified in pig *E. coli* and plasmids found in human and livestock-associated isolates globally, which requires further investigation to understand the full implications of this global dissemination.
[22]	Denmark	Investigation of the clinical epidemiology of all human cases of Livestock-associated methicillin-resistant *Staphylococcus aureus* (LA-MRSA) CC398 blood stream infections (BSIs) during 2010–2015. Cases of LA-MRSA CC398 BSIs were compared to cases of BSIs caused by other types of MRSA and cases of skin and soft tissue infections (SSTI) caused by LA-MRSA CC398. Whole genome sequencing analysis was used to assess the phylogenetic relationship among LA-MRSA CC398 isolates from Danish pigs and cases of BSIs and SSTI.	The number of LA-MRSA CC398 BSIs and SSTIs increased over the years, peaking in 2014, when LA-MRSA CC398 accounted for 16% (7/44) and 21% (211/985) of all MRSA BSIs and SSTIs respectively. Most patients with LA-MRSA CC398 BSI had no contact to livestock, although they tended to live in rural areas. Whole-genome sequence analysis showed that most of the BSI and SSTI isolates were closely related to Danish pig isolates. The study showed that the increasing number of LA-MRSA CC398 BSIs occurred in parallel with a much larger wave of LA-MRSA CC398 SSTIs and an expanding pig reservoir with increased spread into the community and healthcare settings.
[23]	Not applicable	A review of the presence of MRSA along the food chain, and the potential of food producing animals and associated foodstuffs for the transmission of MRSA	LA-MRSA CC398 can be transmitted by direct exposure to animals but indirect exposure is also possible. CC398 isolates are increasingly colonizing people with no contact with livestock, indicating that this lineage is successfully spreading into the community.<br>MRSA introduced into the slaughterhouse on animals or abattoir personnel can lead to contamination of raw meat. Food contamination by human handling may contribute to the spread of MRSA in hospitals and other

			institutional environments. The emergence of MRSA has implications for food safety and surveillance programmes are required for rapid MRSA detection and control.
[24]	Germany	A study reporting the occurrence of an epidemic of LA-MRSA CC398 in Germany in a hospital located in a region characterized by its high livestock density	The first LA-MRSA CC398 was isolated from a screening patient in 2000. After sporadic detections between 2000 and 2004, CC398 accounted for 9.6% of all local MRSA in 2005 and reached a level of 35% in 2013 increasing the burden of MRSA colonization and infections at the hospital.
[25]	Not applicable	The study explored the biochemistry behind reports on AMR in healthcare, agriculture and the environment by focusing on different examples including MRSA, Vancomycin resistant *S. aureus*, colistin resistance and antimicrobial soaps and other products containing disinfectants.	Good stewardship is needed not only in the medical use of antimicrobials, but also for the use of AMs in animal health, the abundant use of AMs in agriculture, and the widespread use of biocides and antiseptics in common household products.
[26]	United States	Whole genome sequencing study to analyze association between *Salmonella* serovars isolated from human, food-animals (swine and poultry) and environments.	The study showed close relationships between *Salmonella* isolates from different hosts, which suggests that animals and the environment are potential sources for dissemination of AMR between *Salmonella* serovars.
[27]	Korea	Investigation of mcr-1-possessing Enterobacteriaceae among Enterobacteriaceae strains from clinical isolates in Korea, and comparison of the genetic details of the plasmids with those in *E. coli* isolates from livestock (10 chicken and one porcine).	Mcr-1-harboring IncI2 plasmids were identified in clinical Enterobacteriaceae isolates. These plasmids were closely associated with those in chicken-origin *E. coli* strains, supporting the concept of mcr-1 dissemination between humans and livestock.
[28]	United Kingdom (UK)	UK One Health report, which includes data on antibiotic use from food-producing animals and humans, data on AMR in bacterial isolates from animals and humans and comparative data on AMR in isolates from retail meat.	The report presents the results of AMR monitoring for key zoonotic and indicator bacterial pathogens for animals and humans: *Campylobacter* spp., non-typhoidal *Salmonella* spp., *E. coli* and LA-MRSA. It gives an overview of available data in the context of the One Health approach.
Presence of resistant bacteria in food products of animal origin			
[29]	United Kingdom	A systematic literature review of data published between 1999 and the end of May 2016 to investigate the occurrence of AMR in bacteria present in pork and poultry meat, dairy products, seafood and fresh produce at retail level.	There was a paucity of AMR data for British produced food. For exporting countries, AMR trends were available mainly from Denmark and the Netherlands. Targeted surveys conducted by the Food Standards Agency at retail level since 2001 provided “snapshots” of AMR in relevant foodborne pathogens (*Salmonella* spp. and *Campylobacter* spp.) in red meat and poultry meat. There is a lack of AMR data on commensal bacteria in food at retail level in the UK.

[30]	Switzerland	A systematic literature review of data published between 1996 and 2016 (covering the Swiss agriculture sector and relevant imported food) to assess AMR bacteria prevalence in retail food and subsequent exposure of Swiss consumers	Raw meat, milk, seafood, and certain fermented dairy products featured a medium to high potential of AMR exposure for Gram-negative and Gram-positive foodborne pathogens and indicator bacteria. There is a need to include food at retail, additional food categories including fermented and novel foods as well as technologically important bacteria and AMR genetics into systematic One Health AMR surveillance to address the observed knowledge gaps identified and enable a comprehensive AMR risk assessment for consumers.
[31]	European Union	The European Union summary report on antimicrobial resistance in zoonotic and indicator bacteria from humans, animals and food in 2017	Resistance data in zoonotic *Salmonella* and *Campylobacter* from humans, animals and food, and resistance in indicator *E. coli* as well as MRSA in animals and food are analyzed and temporal trends assessed.
Association between antimicrobial use in food animals and resistant bacteria in humans			
[32]	United States (US)	Description of the withdrawal of fluoroquinolone use in poultry following an increase of fluoroquinolone resistant *Campylobacter* species in humans	Human infections with fluoroquinolone-resistant *Campylobacter* species became increasingly common after two fluoroquinolones were licensed for use in poultry in 1995 and 1996. This prompted the US Food and Drug Administration to propose the withdrawal of fluoroquinolone use in poultry in 2000. The decision came into effect in 2005.
[33]	Canada	Investigation study to examine the correlation between ceftiofur-resistant *Salmonella* Heidelberg isolated from retail chicken and the incidence of ceftiofur-resistant *Salmonella* Heidelberg infections in humans	There is strong correlation between ceftiofur-resistant *Salmonella enterica* serovar Heidelberg isolated from retail chicken and incidence of ceftiofur-resistant *Salmonella* Heidelberg infections in humans across Canada. Following the voluntary withdrawal of ceftiofur use in hatcheries in Canada in 2005, ceftiofur-resistant *Salmonella* Heidelberg deceased in chickens from 62% to 7% and in humans from 36% to 8%. An increase in resistance levels was observed in humans (from 8% to 12%) and chickens (from 7% to 18%) after reintroduction of its use in young chickens.
[34]	Not applicable	A systematic review and meta-analysis to summarize the effect that interventions to reduce antibiotic use in food-producing animals have on the presence of antibiotic-resistant bacteria in animals and in humans.	Reducing antibiotic use decreased the prevalence of antibiotic-resistant bacteria in animals by about 15% and multidrug-resistant bacteria by 24–32%. The evidence of effect on humans was more limited and less robust, with an association reported mainly in those with direct exposure to food-producing animals. The implications for the general human population are less clear, given

			the low number of studies.
[35]	Not applicable	Modelling study to explore the generic relationship between antibiotic consumption by food animals and the levels of resistant bacterial infections in humans.	The results showed that, for a wide range of scenarios, curtailing the volume of antibiotics consumed by food animals has, as a standalone measure, little impact on the level of resistance in humans. It was also found that reducing the rate of transmission of resistance from animals to humans might be more effective than an equivalent reduction in the consumption of antibiotics in food animals.
[9]	European Union	Described previously	Described previously
Studies with limited evidence of an association between antimicrobial resistance in humans and food-producing animals			
[36]	Denmark	Exposure assessment study to investigate the contribution of different types of meat (broiler, pork and beef) to the exposure of consumers to extended-spectrum beta-lactamase (ESBL) and AmpC beta-lactamases (AmpC) producing *E. coli* and their potential importance for human infections in Denmark.	The overlap between ESBL/AmpC genotypes in meat and human *E. coli* infections was limited, suggesting that meat might constitute a less important source of exposure to humans in Denmark.
[37]	Sweden	An integrated report from the Public Health Agency of Sweden and the National Veterinary Institute that includes data from humans, animals and food.	Presents the results of the monitoring programmes of antibiotic consumption in humans and animals and antibiotic resistance in humans, animals and food.
[5]	Denmark	Described previously	Described previously
[8]	European Union	Described previously	Described previously
[38]	United Kingdom	Prevalence study and types of ESBL-producing and carbapenem-resistant *E. coli* in raw retail beef, chicken, pork, fruit and vegetables in four UK regions (London, East Anglia, the North West, Scotland and Wales).	1.9%, 2.5% and 65.4% of beef, pork and chicken samples were positive for ESBL-producing *E. coli* respectively. 85.6% positive samples from chicken meat carried blaCTX-M-1.blaCTX-M-15, which is dominating in human clinical isolates, was not detected in foodstuffs. None of the fruits or vegetables yielded ESBL-producing *E. coli* and none of the meat, fruit or vegetable samples yielded carbapenem- resistant *E. coli*.
[39]	The Netherlands	Exposure assessment study of ESBL and plasmidic AmpC (pAmpC) producing *E. coli* (EEC) through the consumption of meat from different food animals (cattle, calves, pigs, chickens and lamb) using Quantitative Microbiological Risk Assessment model.	Consumption of beef products led to a higher exposure to EEC than chicken products, although the prevalence of EEC on raw chicken meat was much higher than on beef. The (relative) risk of this exposure for public health is yet unknown due to the lack of a modelling framework and of exposure studies for other potential transmission routes.
[40]	The Netherlands	Pooled analysis study to investigate the molecular relatedness of ESBL/AmpC- producing *E. coli* from humans, animals, food and the	The analysis showed that ESBL-AmpC gene distributions from all reservoirs have a certain level of similarity (i.e. most ESBL/AmpC gene subtypes are

		environment.	found in each individual reservoir). However, most livestock or food-associated reservoirs did not show a high level of similarity in their gene profiles compared with humans from the general and clinical populations. This suggests that livestock reservoirs including poultry and poultry meat are not major contributors to ESBL/AmpC occurrence in humans.
[41]	United Kingdom	Whole genome sequencing study of *Enterococcus faecium* (including Vancomycin-resistant *E. faecium*) from livestock farms (cattle, pig and poultry), retail meat, wastewater and from patients with blood stream infections in the UK.	The majority of *E. faecium* strains infecting patients were largely distinct from those from livestock, with limited sharing of strains and resistance genes.
[42]	England	Whole genome sequencing study to investigate the prevalence and genetic relatedness of *E. coli* isolates from livestock (cattle, pig and poultry), retail meat and patients with bloodstream infections.	There was limited evidence that *E. coli* causing severe human infections had originated from livestock in that region.
Antimicrobial resistance in bacteria linked to food processing practices			
[43]	Not applicable	Investigation study to examine whether exposure and adaptive tolerance to poultry decontaminants could influence resistance to antimicrobials among *Listeria monocytogenes* and *Salmonella enterica* strains.	Increase in resistance to various antibiotics was observed in *L. monocytogenes* and *S. enterica* strains after exposure to sub-inhibitory concentrations of poultry decontaminants (especially acidified sodium chlorite). Major differences were observed between the strains tested; suggesting that changes in the pattern of susceptibility to antibiotics after exposure to decontaminants might be strain specific rather than species or genus specific.
[44]	Not applicable	Investigation of the tolerance of a collection of susceptible and multidrug-resistant *Salmonella enterica* strains to a panel of seven commercially available food-grade biocide formulations	Exposure to sublethal concentrations of individual active biocidal agents can lead to the emergence of tolerant isolates of *S. enterica.* This emergence was associated with changes in antimicrobial susceptibilities.
[45]	Canada	A review highlighting a variety of bacterial stress responses that have been linked to AMR	The exposure of bacteria to different stresses such as nutrient starvation/limitation (nutrient stress), reactive oxygen and nitrogen species (oxidative/ nitrosative stress), membrane damage (envelope stress), elevated temperature (heat stress) and ribosome disruption (ribosomal stress), all impact bacterial susceptibility to a variety of AMs through their initiation of stress responses that positively impact recruitment of resistance determinants or promote physiological changes that compromise antimicrobial activity.

[46]	Not applicable	A review on the impact of food processing on the transfer of AMR to humans	Food processes that kill bacteria in food products decrease the risk of transmission of antimicrobial resistance. Raw food products can be consumed without having undergone prior processing or preservation and therefore hold a higher risk for transfer of AMR to humans. Under minimal processing or preservation treatment conditions, sub-lethally damaged or stressed cells can be maintained in the food inducing AMR build-up and increasing the risk of resistance transfer.
[47]	Denmark	Study examining the prevalence of biocide resistant *Salmonella* spp. in Danish pig slaughterhouses to evaluate if there was a correlation between susceptibilities to biocides and antibiotics, and to examine if cleaning and disinfection select isolates with changed susceptibility toward biocides or antibiotics	No resistance was found toward the biocides tested, but the isolates obtained after cleaning had higher minimum inhibitory concentration values toward one of the disinfectants (Incimaxx DES) compared to isolates obtained before cleaning and disinfection. This could indicate selection of strains that are more tolerant, due to the cleaning and disinfection. A weak correlation was observed between susceptibility to biocides and some AMs.
[25]	Not applicable	Described previously	Described previously
[30]	Switzerland	Described previously	Described previously

References:

1. Aarestrup, F.M.; Seyfarth, A.M.; Emborg, H.D.; Pedersen, K.; Hendriksen, R.S.; Bager, F. Effect of abolishment of the use of antimicrobial agents for growth promotion on occurrence of antimicrobial resistance in fecal Enterococci from food animals in Denmark. *Antimicrob. Agents Chemother.* **2001**, *45*, 2054–2059.
2. Hammerum, A.M.; Heuer, O.E.; Emborg, H.-D.; Bagger-Skjøt, L.; Jensen, V.F.; Rogues, A.-M.; Skov, R.L.; Agersø, Y.; Brandt, C.T.; Seyfarth, A.M., et al. Danish integrated antimicrobial resistance monitoring and research program. *Emerg. Infect. Dis.* **2007**, *13*, 1632–1639.
3. Agersø, Y.; Aarestrup, F.M. Voluntary ban on cephalosporin use in Danish pig production has effectively reduced extended-spectrum cephalosporinase-producing Escherichia coli in slaughter pigs. *J. Antimicrob. Chemother.* **2013**, *68*, 569–572.
4. Chantziaras, I.; Boyen, F.; Callens, B.; Dewulf, J. Correlation between veterinary antimicrobial use and antimicrobial resistance in food-producing animals: A report on seven countries. *J.Antimicrob. Chemother.* **2013**, *69*, 827–834.
5. DANMAP. Use of antimicrobial agents and occurrence of antimicrobial resistance in bacteria from food animals, food and humans in denmark. 2015 Available online: https://www.danmap.org/-/media/arkiv/projekt-sites/danmap/danmap-reports/danmap--2015/danmap-2015.pdf?la=en (accessed on: 15 May 2017).
6. Dorado-Garcia, A.; Mevius, D.J.; Jacobs, J.J.; Van Geijlswijk, I.M.; Mouton, J.W.; Wagenaar, J.A.; Heederik, D.J. Quantitative assessment of antimicrobial resistance in livestock during the course of a nationwide antimicrobial use reduction in the Netherlands. *J. Antimicrobe. Chemother.* **2016**, *71*, 3607–3619.
7. McCrackin, M.A.; Helke, K.L.; Galloway, A.M.; Poole, A.Z.; Salgado, C.D.; Marriott, B.P. Effect of antimicrobial use in agricultural animals on drug-resistant foodborne campylobacteriosis in humans: A systematic literature review. *Crit. Rev. Food Sci. Nutr.* **2016**, *56*, 2115–2132.
8. ECDC/EFSA/EMA. First joint report on the integrated analysis of the consumption of antimicrobial agents and occurrence of antimicrobial resistance in bacteria from humans and food-producing animals. *Efsa Journal* **2015**, *13*, 37–45.
9. ECDC/EFSA/EMA. Second joint report on the integrated analysis of the consumption of antimicrobial agents and occurrence of antimicrobial resistance in bacteria from humans and food-producing animals: Joint interagency antimicrobial consumption and resistance analysis (jiacra) report. *Efsa Journal* **2017**, *15*.
10. MARAN. Monitoring of antimicrobial resistance and antibiotic usage in animals in the netherlands in 2016 Available online: https://www.wur.nl/upload_mm/6/9/5/4f37c335-224c-4595-82e4-be6182c0a5e1_74ce6009-b112-428d-aeb7-99b95063aab6_Maran%20report%202017.pdf (accessed on: 14 December 2017).
11. Callens, B.; Cargnel, M.; Sarrazin, S.; Dewulf, J.; Hoet, B.; Vermeersch, K.; Wattiau, P.; Welby, S. Associations between a decreased veterinary antimicrobial use and resistance in commensal Escherichia coli from Belgian livestock species (2011-2015). *Prev. Vet. Med.* **2018**, *157*, 50–58.
12. Armand-Lefevre, L.; Ruimy, R.; Andremont, A. Clonal comparison of Staphylococcus aureus isolates from healthy pig farmers, human controls, and pigs. *Emerg. Infect. Dis.* **2005**, *11*, 711–714.
13. Hald, T.; Lo Fo Wong, D.M.; Aarestrup, F.M. The attribution of human infections with antimicrobial resistant Salmonella bacteria in Denmark to sources of animal origin. *Foodborne Pathog. Dis.* **2007**, *4*, 313–326.
14. Mather, A.E.; Reid, S.W.; Maskell, D.J.; Parkhill, J.; Fookes, M.C.; Harris, S.R.; Brown, D.J.; Coia, J.E.; Mulvey, M.R.; Gilmour, M.W.; et al. Distinguishable epidemics of multidrug-resistant Salmonella typhimurium dt104 in different hosts. *Science* **2013**, *341*, 1514–1517.
15. Hasman, H.; Hammerum, A.M.; Hansen, F.; Hendriksen, R.S.; Olesen, B.; Agersø, Y.; Zankari, E.; Leekitcharoenphon, P.; Stegger, M.; Kaas, R.S.; et al. Detection of mcr-1 encoding plasmid-mediated colistin-resistant Escherichia coli isolates from human bloodstream infection and imported chicken meat, Denmark 2015. *Euro. Surveill.* **2015**, *20*, 30085.
16. Lazarus, B.; Paterson, D.L.; Mollinger, J.L.; Rogers, B.A. Do human extraintestinal Escherichia coli infections resistant to expanded-spectrum cephalosporins originate from food-producing animals? A systematic review. *Clin. Infect. Dis.* **2015**, *60*, 439–452.
17. Anjum, M.F.; Duggett, N.A.; AbuOun, M.; Randall, L.; Nunez-Garcia, J.; Ellis, R.J.; Rogers, J.; Horton, R.; Brena, C.; Williamson, S.; et al. Colistin resistance in Salmonella and Escherichia coli isolates from a pig farm in Great Britain. *J. Antimicrob. Chemother.* **2016**, *71*, 2306–2313.

18. European Medicines Agency. Updated advice on the use of colistin products in animals within the european union: Development of resistance and possible impact on human and animal health. 2016 Available online: https://www.ema.europa.eu/en/documents/scientific-guideline/updated-advice-use-colistin-products-animals-within-european-union-development-resistance-possible_en-0.pdf (accessed on: 25 April 2019).
19. Liu, Y.Y.; Wang, Y.; Walsh, T.R.; Yi, L.X.; Zhang, R.; Spencer, J.; Doi, Y.; Tian, G.; Dong, B.; Huang, X.; et al. Emergence of plasmid-mediated colistin resistance mechanism mcr-1 in animals and human beings in china: A microbiological and molecular biological study. *Lancet Infect. Dis.* **2016**, *16*, 161–168.
20. Carroll, L.M.; Wiedmann, M.; den Bakker, H.; Siler, J.; Warchocki, S.; Kent, D.; Lyalina, S.; Davis, M.; Sischo, W.; Besser, T.; et al. Whole-genome sequencing of drug-resistant Salmonella enterica isolates from dairy cattle and humans in New York and Washington states reveals source and geographic associations. *Appl. Environ. Microbiol.* **2017**, *83*.
21. Duggett, N.A.; Sayers, E.; AbuOun, M.; Ellis, R.J.; Nunez-Garcia, J.; Randall, L.; Horton, R.; Rogers, J.; Martelli, F.; Smith, R.P.; et al. Occurrence and characterization of mcr-1-harbouring Escherichia coli isolated from pigs in Great Britain from 2013 to 2015. *J. Antimicrob. Chemother.* **2017**, *72*, 691–695.
22. Larsen, J.; Petersen, A.; Larsen, A.R.; Sieber, R.N.; Stegger, M.; Koch, A.; Aarestrup, F.M.; Price, L.B.; Skov, R.L. Emergence of livestock-associated methicillin-resistant Staphylococcus aureus bloodstream infections in Denmark. *Clin. Infect. Dis.* **2017**, *65*, 1072–1076.
23. Oniciuc, E.-A.; Nicolau, A.I.; Hernández, M.; Rodríguez-Lázaro, D. Presence of methicillin-resistant Staphylococcus aureus in the food chain. *Trends Food Sci.Technol.* **2017**, *61*, 49–59.
24. van Alen, S.; Ballhausen, B.; Peters, G.; Friedrich, A.W.; Mellmann, A.; Kock, R.; Becker, K. In the centre of an epidemic: Fifteen years of LA-MRSA CC398 at the university Hospital Munster. *Vet. Microbiol.* **2017**, *200*, 19–24.
25. Venter, H.; Henningsen, M.L.; Begg, S.L. Antimicrobial resistance in healthcare, agriculture and the environment: The biochemistry behind the headlines. *Essays Biochem.* **2017**, *61*, 1–10.
26. Pornsukarom, S.; van Vliet, A.H.M.; Thakur, S. Whole genome sequencing analysis of multiple Salmonella serovars provides insights into phylogenetic relatedness, antimicrobial resistance, and virulence markers across humans, food animals and agriculture environmental sources. *BMC Genom.* **2018**, *19*, 801.
27. Yoon, E.J.; Hong, J.S.; Yang, J.W.; Lee, K.J.; Lee, H.; Jeong, S.H. Detection of mcr-1 plasmids in enterobacteriaceae isolates from human specimens: Comparison with those in Escherichia coli isolates from livestock in Korea. *Ann. Lab. Med.* **2018**, *38*, 555–562.
28. Veterinary Medicines Directorate. UK one health report—joint report on antibiotic use and antibiotic resistance, 2013–2017. New Haw, Addlestone: Veterinary medicines directorate. Available online: https://assets.publishing.service.gov.uk/government/uploads/system/uploads/attachment_data/file/775075/One_Health_Report_2019_v45.pdf (accessed date: 25 April 2019).
29. Mateus, A.; Takahashi, E.A.; Abdelrazeq Elkholly, D.; Crotta, M.; Ekiri, A.; Wylie, C.; Guinat, C.; Patricio, M.; Marshall, L.; Pinto Ferreira, J.; et al. *A systematic review to assess the significance of the food chain in the context of antimicrobial resistance with particular respect to retail pork, poultry meat, dairy products, seafood and fresh produce in the UK*. Technical Report; Royal Veterinary College, University of London, UK, November 2016.
30. Jans, C.; Sarno, E.; Collineau, L.; Meile, L.; Stark, K.D.C.; Stephan, R. Consumer exposure to antimicrobial resistant bacteria from food at swiss retail level. *Front. Microbiol.* **2018**, *9*, 362.
31. EFSA/ECDC. The european union summary report on antimicrobial resistance in zoonotic and indicator bacteria from humans, animals and food in 2017. *Efsa Journal* **2019**, *17*, 1–21.
32. Nelson, J.M.; Chiller, T.M.; Powers, J.H.; Angulo, F.J. Fluoroquinolone-resistant campylobacter species and the withdrawal of fluoroquinolones from use in poultry: A public health success story. *Clin. Infect.dis.* **2007**, *44*, 977–980.
33. Dutil, L.; Irwin, R.; Finley, R.; Ng, L.K.; Avery, B.; Boerlin, P.; Bourgault, A.M.; Cole, L.; Daignault, D.; Desruisseau, A.; et al. Ceftiofur resistance in Salmonella enterica serovar heidelberg from chicken meat and humans, canada. *Emerg. Infect. Dis.* **2010**, *16*, 48–54.
34. Tang, K.L.; Caffrey, N.P.; Nobrega, D.B.; Cork, S.C.; Ronksley, P.E.; Barkema, H.W.; Polachek, A.J.; Ganshorn, H.; Sharma, N.; Kellner, J.D.; et al. Restricting the use of antibiotics in food-producing animals and its associations with antibiotic resistance in food-producing animals and human beings: A systematic review and meta-analysis. *Lancet Planet. Health* **2017**, *1*, e316–e327.
35. van Bunnik, B.A.D.; Woolhouse, M.E.J. Modelling the impact of curtailing antibiotic usage in food animals on antibiotic resistance in humans. *R. Soc. Open Sci.* **2017**, *4*, 161067–161067.

36. Carmo, L.P.; Nielsen, L.R.; da Costa, P.M.; Alban, L. Exposure assessment of extended-spectrum beta-lactamases/ampc beta-lactamases-producing Escherichia coli in meat in Denmark. *Infect. Ecol. Epidemiol.* **2014**, *4*, 22924.
37. SWEDRES-SVARM. Consumption of antibiotics and occurence of antibiotic resistance in sweden. Uppsala, sweden. 2014 Available online: https://www.sva.se/globalassets/redesign2011/pdf/om_sva/publikationer/swedres_svarm2014.pdf (accessed on: 15 May 2017).
38. Randall, L.P.; Lodge, M.P.; Elviss, N.C.; Lemma, F.L.; Hopkins, K.L.; Teale, C.J.; Woodford, N. Evaluation of meat, fruit and vegetables from retail stores in five United Kingdom regions as sources of extended-spectrum beta-lactamase (esbl)-producing and carbapenem-resistant Escherichia coli. *Int. J. Food Microbiol.* **2017**, *241*, 283–290.
39. Evers, E.G.; Pielaat, A.; Smid, J.H.; van Duijkeren, E.; Vennemann, F.B.C.; Wijnands, L.M.; Chardon, J.E. Comparative exposure assessment of esbl-producing Escherichia coli through meat consumption. *PLoS ONE* **2017**, *12*, e0169589.
40. Dorado-Garcia, A.; Smid, J.H.; van Pelt, W.; Bonten, M.J.M.; Fluit, A.C.; van den Bunt, G.; Wagenaar, J.A.; Hordijk, J.; Dierikx, C.M.; Veldman, K.T.; et al. Molecular relatedness of esbl/ampc-producing Escherichia coli from humans, animals, food and the environment: A pooled analysis. *J. Antimicrobe. Chemother.* **2018**, *73*, 339–347.
41. Gouliouris, T.; Raven, K.E.; Ludden, C.; Blane, B.; Corander, J.; Horner, C.S.; Hernandez-Garcia, J.; Wood, P.; Hadjirin, N.F.; Radakovic, M.; et al. Genomic surveillance of Enterococcus faecium reveals limited sharing of strains and resistance genes between livestock and humans in the United Kingdom. *mBio* **2018**, *9*, e01780–e01718.
42. Ludden, C.; Raven, K.E.; Jamrozy, D.; Gouliouris, T.; Blane, B.; Coll, F.; de Goffau, M.; Naydenova, P.; Horner, C.; Hernandez-Garcia, J., et al. One health genomic surveillance of Escherichia coli demonstrates distinct lineages and mobile genetic elements in isolates from humans versus livestock. *mBio* **2019**, *10*, e02693–e02618.
43. Alonso-Hernando, A.; Capita, R.; Prieto, M.; Alonso-Calleja, C. Comparison of antibiotic resistance patterns in Listeria monocytogenes and Salmonella enterica strains pre-exposed and exposed to poultry decontaminants. *Food Control* **2009**, *20*, 1108–1111.
44. Condell, O.; Iversen, C.; Cooney, S.; Power, K.A.; Walsh, C.; Burgess, C.; Fanning, S. Efficacy of biocides used in the modern food industry to control Salmonella enterica, and links between biocide tolerance and resistance to clinically relevant antimicrobial compounds. *Appl. Environ. Microbiol.* **2012**, *78*, 3087–3097.
45. Poole, K. Bacterial stress responses as determinants of antimicrobial resistance. *J. Antimicrob. Chemother.* **2012**, *67*, 2069–2089.
46. Verraes, C.; Van Boxstael, S.; Van Meervenne, E.; Van Coillie, E.; Butaye, P.; Catry, B.; de Schaetzen, M.A.; Van Huffel, X.; Imberechts, H.; Dierick, K.; et al. Antimicrobial resistance in the food chain: A review. *Int. J. Environ. Res. Public Health* **2013**, *10*, 2643–2669.
47. Gantzhorn, M.R.; Pedersen, K.; Olsen, J.E.; Thomsen, L.E. Biocide and antibiotic susceptibility of Salmonella isolates obtained before and after cleaning at six Danish pig slaughterhouses. *Int. J. Food Microbiol.* **2014**, *181*, 53–59.

© 2020 by the authors. Submitted for possible open access publication under the terms and conditions of the Creative Commons Attribution (CC BY) license (http://creativecommons.org/licenses/by/4.0/).
